# Association of Stage Shift and Population Mortality Among Patients With Non–Small Cell Lung Cancer

**DOI:** 10.1001/jamanetworkopen.2021.37508

**Published:** 2021-12-17

**Authors:** Raja Flores, Parth Patel, Naomi Alpert, Bruce Pyenson, Emanuela Taioli

**Affiliations:** 1 Department of Thoracic Surgery, Icahn School of Medicine at Mount Sinai, Mount Sinai Health System, New York, New York; 2 Institute for Translational Epidemiology and Tisch Cancer Institute, Icahn School of Medicine at Mount Sinai, New York, New York; 3 NYU School of Global Public Health, New York, New York; 4 Milliman Inc, New York, New York

## Abstract

**Importance:**

Early detection by computed tomography and a more attention-oriented approach to incidentally identified pulmonary nodules in the last decade has led to population stage shift for non–small cell lung cancer (NSCLC). This stage shift could substantially confound the evaluation of newer therapeutics and mortality outcomes.

**Objective:**

To investigate the association of stage shift with population mortality among patients with NSCLC.

**Design, Setting, and Participants:**

This retrospective cohort study was performed from October 2020 to June 2021 and used data from the Surveillance, Epidemiology, and End Results (SEER) registries to assess all patients from 2006 to 2016 with NSCLC.

**Main Outcomes and Measures:**

Incidence-based mortality was evaluated by year-of-death. To assess shifts in diagnostic characteristics, clinical stage and histology distributions were examined by year using χ^2^ tests. Trends were assessed using the average annual percentage change (AAPC), calculated with JoinPoint software. Kaplan-Meier survival analysis assessed overall survival according to stage and compared those missing any stage with those with a reported stage.

**Results:**

The final sample contained 312 382 patients; 166 657 (53.4%) were male, 38 201 (12.2%) were Black, and 249 062 (79.7%) were White; the median (IQR) age was 68 (60-76) years; 163 086 (52.2%) had adenocarcinoma histology. Incidence-based mortality within 5 years of diagnosis decreased from 2006 to 2016 (AAPC, −3.7; 95% CI, −4.1 to −3.4). When assessing stage shift, there was significant association between year-of-diagnosis and clinical stage, with stage I/II diagnosis increasing from 26.5% to 31.2% (AAPC, 1.5; 95% CI, 0.5 to 2.5); and stage III/IV diagnosis decreasing significantly from 70.8% to 66.1% (AAPC, −0.6; 95% CI, −1.0 to −0.2). Missing staging information was not associated with year-of-diagnosis (AAPC, −1.6; 95% CI, −7.4 to 4.5). Year-of-diagnosis was significantly associated with tumor histology (χ^2^ = 8990.0; *P* < .001). There was a significant increase in adenocarcinomas: 42.9% in 2006 to 59.0% in 2016 (AAPC, 3.4; 95% CI, 2.9 to 3.9). Median (IQR) survival for stage I/II was 57 months (18 months to not reached); stage III/IV was 7 (2-19) months; and missing stage was 10 (2-28) months. When compared with those with known stage, those without stage information had significantly worse survival than those with stage I/II, with survival between those with stage III and stage IV (log-rank χ^2^ = 87 125.0; *P* < .001).

**Conclusions and Relevance:**

This cohort study found an association between decreased mortality and a corresponding diagnostic shift from later to earlier stage. These findings suggest that studies investigating the effect of treatment on lung cancer must take into account stage shift and the confounding association with survival and mortality outcome.

## Key Points

**Question:**

To what extent does stage shift act as a confounding variable in the evaluation of population mortality of non–small cell lung cancer?

**Findings:**

In this cohort study of 312 382 patients, stage shift from later to earlier stage disease over the last decade was associated with improved mortality among people with lung cancer.

**Meaning:**

These findings suggest that studies investigating treatments for lung cancer must take into account stage shift and the confounding association with survival and mortality outcome.

## Teaser

This cohort study investigates the association of stage shift with population mortality among patients diagnosed with non–small cell lung cancer between 2006 and 2016.

## Introduction

Lung cancer remains among the leading causes of cancer death in the United States.^1,2^ Based on the Annual Report to the Nation, incidence rates for lung cancer have significantly declined from 2012 to 2016 (average annual percent change [AAPC] of −2.6% for male individuals and −1.1% for female individuals).^1^ Moreover, mortality for lung cancer from 2013 to 2017 has decreased at a greater rate compared with the incidence and has experienced one of the largest declines in death rates compared with other common cancer deaths (AAPC of −4.8% among male individuals and −3.7% among female individuals).^1^ The improved outcomes with lung cancer are quite multifactorial and can be attributed to advances in prevention, early detection, and treatment of lung cancer.^3,4^

In recent years, the many advances in medical therapeutics, such as targeted therapy, immunotherapy, and chemotherapy, have garnered interest in the role they may play in affecting lung cancer population-level mortality.^5,6^ In particular, Howlader et al,^5^ using data from Surveillance, Epidemiology, and End Results (SEER) registries from 2001 to 2016 concluded that the observed lung cancer mortality during the study timeframe was likely to be explained by the approval and use of targeted therapies especially for non–small cell lung cancer (NSCLC). However, Howlader et al^5^ do not provide direct evidence to support the direct effect of targeted therapies in affecting mortality but rather cite how the lack of other major advances in prevention or treatment explain the substantial decline in incidence-based mortality.

Many randomized clinical trials have demonstrated the significant survival benefit of targeted therapies among patients harboring driver variants among the druggable oncogenes, such as epidermal growth factor receptor (EGFR) and anaplastic lymphoma kinase (ALK).^7,8,9^ Moreover, immune-based therapies, in particular programmed cell death protein 1–programmed death ligand 1 (PD-1–PD-L1) inhibitors have substantially improved survival among patients with NSCLC even if they do not carry EGFR and ALK variants.^10,11,12,13^ Since 20% of patients with NSCLC have a substantial and sustained response to targeted and immunotherapies, these therapies certainly play a role in affecting population mortality.^14^ Nevertheless, in associating the role of targeted and immunotherapies in decreasing mortality, Howlader et al^5^ minimize the role of early detection and stage shift on mortality by suggesting that “patients moved from unknown stages to more specific stage categories (as a result of the availability of better imaging) rather than shifting from late to early stages.”^5^ However, the role of stage shift due to early detection in affecting NSCLC population-level mortality may not be as minimal as suggested. As a matter of fact, computed tomography (CT) screening for lung cancer is the only modality demonstrating decreased disease-specific mortality. As per the National Lung Screening Trial (NLST), screening with low-dose CT resulted in a positive screening rate of 24.2% compared with 6.9% with chest radiography.^6^ The increased rate of early detection has translated into decreased mortality among patients diagnosed with lung cancer. The NLST, Nederlands–Leuvens Longkanker Screenings Onderzoek (NELSON) trial, and International Early Lung Cancer Action Project (I-ELCAP) have demonstrated that CT screening can identify early stage disease in 4 of 5 patients unknowingly harboring lung cancer^6,15,16^_._ Given these survival benefits, the United States Preventive Services Task Force (USPSTF) has recommended since 2013 annual screening with low-dose CT as standard of care with further expansion of the screening criteria in 2021 to include younger adults with a smaller pack-year smoking history in order increase early detection and subsequently decrease mortality from lung cancer.^17^

To better understand the association of early detection with lung cancer mortality, it is pertinent to evaluate the extent of stage shift in the last decade and its effect in contributing to NSCLC incidence-based mortality. We hypothesize that lung cancer early detection by CT, both intentional and nonintentional (back-alley screening), such as cardiac CT angiograms screening for coronary disease, and a more attention-oriented approach to incidentally identified pulmonary nodules, is associated with a stage shift and subsequent decreased mortality from earlier surgical intervention. We performed a more systematic exhaustive stage and histology evaluation over the same period (2006-2016) using the same SEER data set and methodology as Howlader et al.^5^

## Methods

### Data Source and Study Selection

The SEER Program compiles information on cancer incidence, including patient demographics, self-reported race and ethnicity, tumor characteristics, treatment, and vital status from population-based cancer-registries.^18^ The registries included in SEER currently cover approximately 35% of the United States population.^18^ This retrospective cohort study analysis was conducted from October 1, 2020, to June 30, 2021, using data extracted from SEER. The study followed the Strengthening the Reporting of Observational Studies in Epidemiology (STROBE) reporting guideline.^19^ Since the SEER database is public and deidentified, the study was deemed as exempt research by the institutional review board at the Icahn School of Medicine at Mount Sinai and informed consent was waived.

Based on when the International Early Lung Cancer Action Program began recommending lung cancer screening,^16^ SEER 18 registries were queried for patients from 2006 to 2016 with microscopically confirmed lung cancer (n = 502 583), where lung cancer was the first or only primary malignant neoplasm (n = 375 429) and was not reported on autopsy or death certificate (n = 374 855). Analysis was limited to those with non–small cell histology for a final sample of 312 382.

### Statistical Analysis

Clinical stage and histology distributions were examined by year to assess shifts in diagnostic characteristics over time using χ^2^ tests. Additionally, the AAPC in tumor characteristics over time was calculated. Clinical stage was based on the American Joint Committee on Cancer sixth (2006-2015) edition and combined SEER stage group (2016). Because detailed stage information was missing for 6.3% of the sample, SEER historic staging was used to impute stage for these patients. Those with historic stage values of localized were coded as stage I/II, regional was coded as stage III, and distant was coded as stage IV. A sensitivity analysis was conducted through 2015, before the staging change in SEER and found similar results to what is presented. Histology was classified as squamous cell carcinoma, adenocarcinoma, or other NSCLC, using *International Classification of Diseases for Oncology Third Edition (ICD-O-3)* codes adopted from Egevad et al^20^ and based on guidelines from the International Agency for Research on Cancer. In order to further characterize those patients missing any staging information, first course of treatment was examined, as surgical resection would reflect standard of care for early-stage tumors. Median survival was calculated according to stage, and Kaplan-Meier curves were used to compare those missing any stage with those with a reported stage. The AAPC was calculated using Joinpoint software version 4.9.0.0 (National Cancer Institute), other analyses were conducted using SAS software version 9.4 (SAS Institute).^21^ Incidence-based mortality within 5 years of diagnosis, which was defined as the number of deaths among those with a NSCLC diagnosis in SEER, divided by the total population residing in the geographic areas of the SEER registries, was calculated in SEER*Stat for 2006 to 2016.^22^ Figures were created in R version 3.4.0 (R Project for Statistical Computing). The significance threshold was *P* < .05. Significance was based on χ^2^ tests (for the association between characteristics and year), and *t* tests for the joinpoint trends. All testing was 2-sided. Statistical analysis was performed from October 2020 to June 2021.

## Results

There were 312 382 patients in SEER diagnosed with NSCLC during 2006 to 2016. Among these patients in the study, 166 657 (53.4%) were male, 38 201 (12.2%) were Black, and 249 062 (79.7%) were White; the median (IQR) age was 68 (60-76) years. Year of diagnosis was evenly distributed among the sample (approximately 9% diagnosed in each year of study). There were 88 179 patients (28.2%) diagnosed at stage I/II and 217 037 (69.5%) at stage III/IV; 7166 (2.3%) were missing any staging information. The majority of patients had adenocarcinoma histology (52.2% [n = 163 086]); 20.3% (n = 63 451) of patients were missing information about their tumor size; 13.3% (n = 41 610) had a tumor size less than 2 cm, whereas 26.9% (n = 84 150) had tumor size greater than or equal to 5 cm (Table).

**Table.** Demographics and Clinical Characteristics of the Population Under Study

Variable	Patients, No. (%)
Age, median (IQR), y	68 (60-76)
Sex	
Male	166 657 (53.4)
Female	145 725 (46.7)
Race and ethnicity	
Black	38 201 (12.2)
White	249 062 (79.7)
Other^a^	24 345 (7.8)
Missing	774 (0.3)
Year of diagnosis	
2006	28 798 (9.2)
2007	28 620 (9.2)
2008	28 827 (9.2)
2009	28 685 (9.2)
2010	28 232 (9.0)
2011	28 118 (9.0)
2012	27 883 (8.9)
2013	27 999 (9.0)
2014	28 167 (9.0)
2015	28 570 (9.2)
2016	28 483 (9.1)
Stage	
I/II	88 179 (28.23)
III/IV	217 037 (69.5)
Missing	7166 (2.3)
Histology	
Squamous cell carcinoma	81 948 (26.2)
Adenocarcinoma	163 086 (52.2)
Other NSCLC	67 348 (21.6)
Tumor size, cm	
<1	4818 (1.5)
1-1.9	36 792 (11.8)
2-2.9	48 518 (15.5)
3-3.9	41 739 (13.4)
4-4.9	32 914 (10.5)
≥5	84 150 (26.9)
Missing	63 451 (20.3)

Abbreviation: NSCLC, non–small cell lung cancer.

^a^ Other races in the Surveillance, Epidemiology, and End Results (SEER) Program included the following: American Indian, Alaskan Native, and Asian/Pacific Islander.

**Figure 1.**
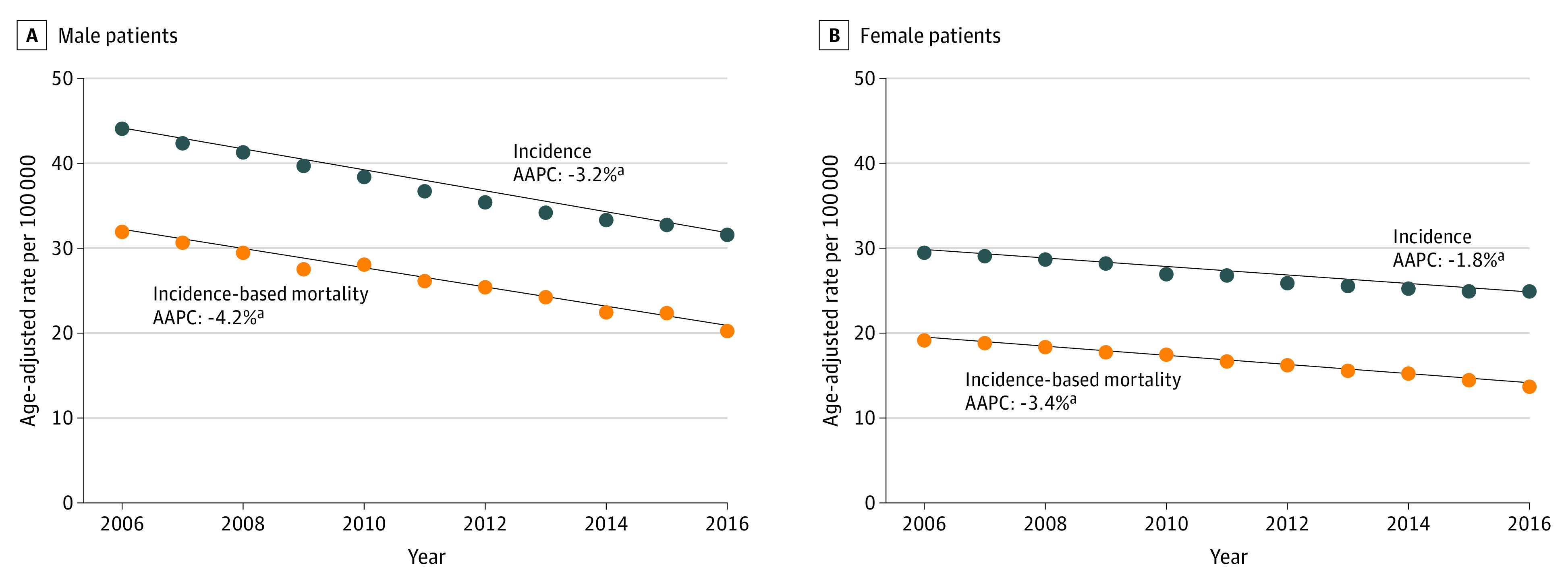
Incidence and Incidence-Based Mortality for Non–Small Cell Lung Cancer According to Sex From 2006-2016 AAPC indicates average annual percent change. ^a^This AAPC value is statistically significant.

Incidence and incidence-based mortality for NSCLC according to sex are presented in Figure 1. The decline in the incidence for NSCLC was greater for male individuals (AAPC, −3.2; 95% CI, −3.6 to −2.9) compared with female individuals (AAPC, −1.8; 95% CI, −2.3 to −1.3) from 2006 to 2016. Incidence-based mortality for both male and female individuals declined at a greater rate compared with the incidence. For male individuals, incidence-based mortality AAPC from 2006 to 2016 was −4.2 (95% CI, −4.6 to −3.7), whereas for female individuals it was −3.4 (95% CI, −3.9 to −2.9).

**Figure 2.**
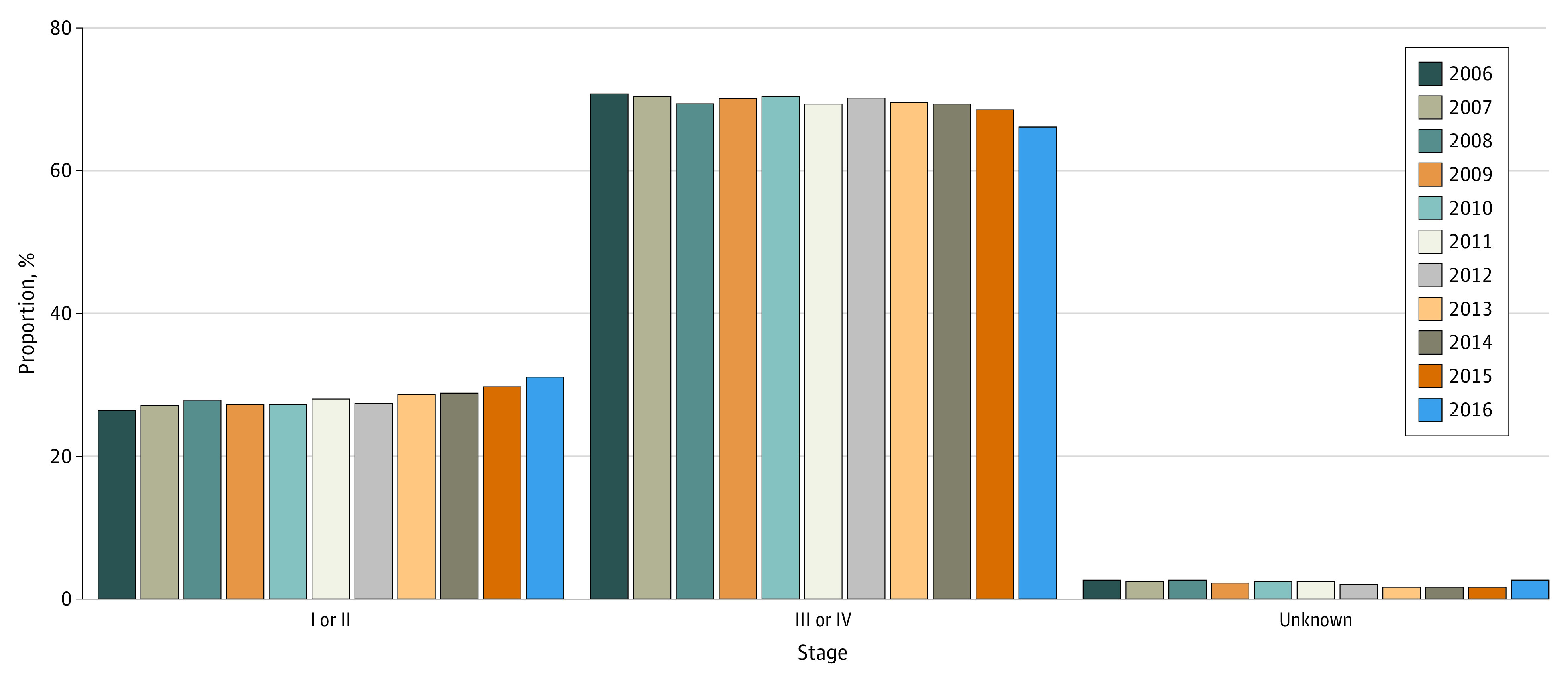
Shift in Stage at Diagnosis Over Time, 2006-2016

There was a significant association between year of diagnosis and stage, with the percentage in each year diagnosed at stage I/II increasing from 26.5% to 31.2% from 2006 to 2016, corresponding to a statistically significant AAPC of 1.5 (95% CI, 0.5 to 2.5); the percentage of patients diagnosed at stage III/IV decreased significantly from 70.8% to 66.1% (AAPC, −0.6; 95% CI, −1.0 to −0.2). The percentage missing staging information did not significantly change from 2006 (2.8%) to 2015 (1.7%) (AAPC, 95% CI, −1.6; −7.4 to 4.5) (Figure 2). A sensitivity analysis was conducted through 2015, before the staging change in SEER and found similar results.

**Figure 3.**
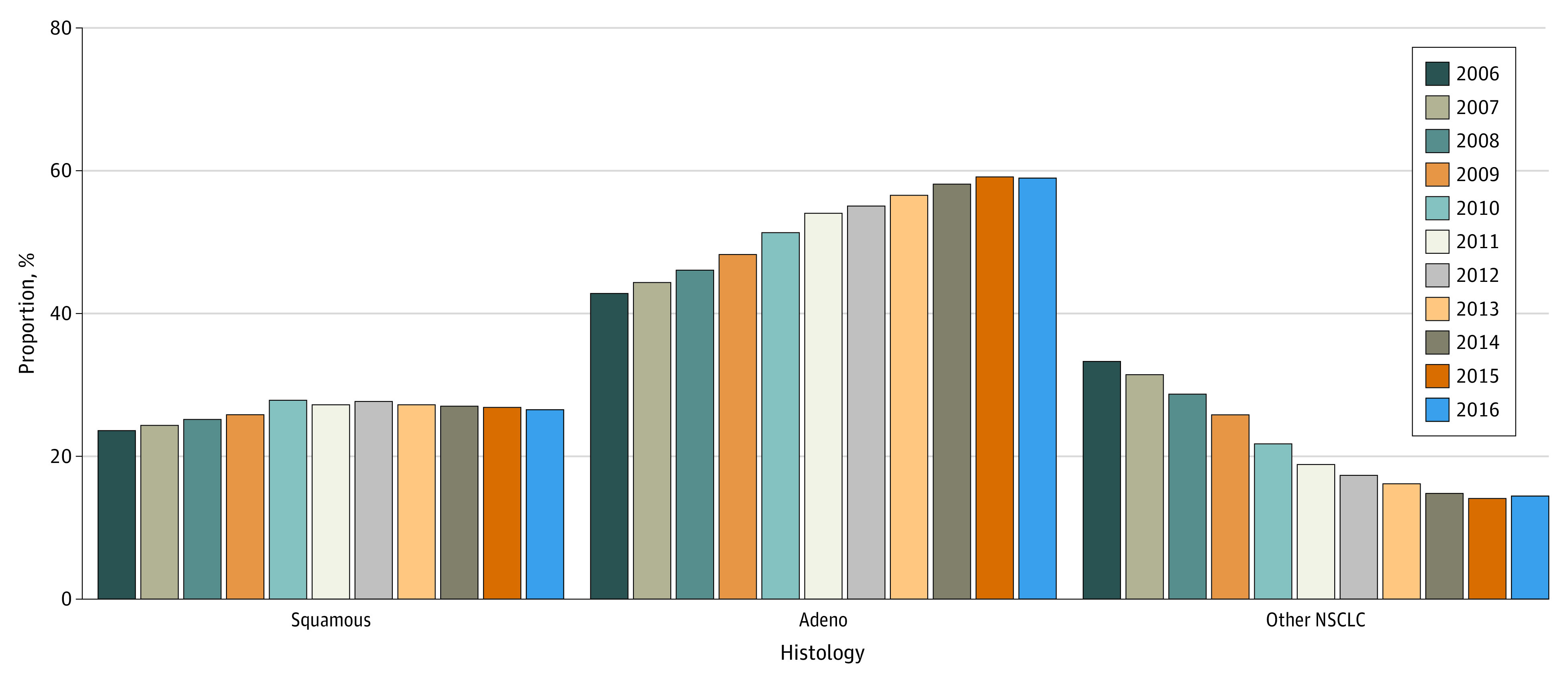
Shift in Histology Over Time, 2006-2016 NSCLC indicates non–small cell lung cancer.

Year of diagnosis was significantly associated with tumor histology (χ^2^ = 8990.0; *P* < .001). There was a large, statistically significant increase in those diagnosed with adenocarcinomas, from 42.9% in 2006 to 59.0% in 2016 (AAPC, 3.4; 95% CI, 2.9 to 3.9). There was also a significant increase in squamous cell carcinoma, from 23.7% in 2006 to 26.0% in 2016 (AAPC, 1.2; 95% CI, 1.1 to 1.4), and a significant decrease in those diagnosed with other NSCLC histologies from 33.4% in 2006 to 14.4% in 2016 (AAPC, −8.4; 95% CI, −10.5 to −6.4) (Figure 3).

Those missing any staging information had a mean (SD) age of 71.5 (0.1) years at diagnosis. They were majority male (51.8%) and White (77.5%). Generally, those missing stage were more highly represented in earlier years. Fewer patients than in the overall group had adenocarcinoma (41.9%); 26.8% (n = 1921) had squamous cell histology and 31.3% (n = 2246) had other NSCLC histologies. The majority of patients (78.8%; n = 5718) were missing tumor size. Among patients without stage information, 86.7% (n = 6216) did not receive surgery to the primary site as part of their first course of treatment; 20.4% (n = 1463) were confirmed to have received chemotherapy; and 13.4% (n = 960) received external beam radiotherapy.

**Figure 4.**
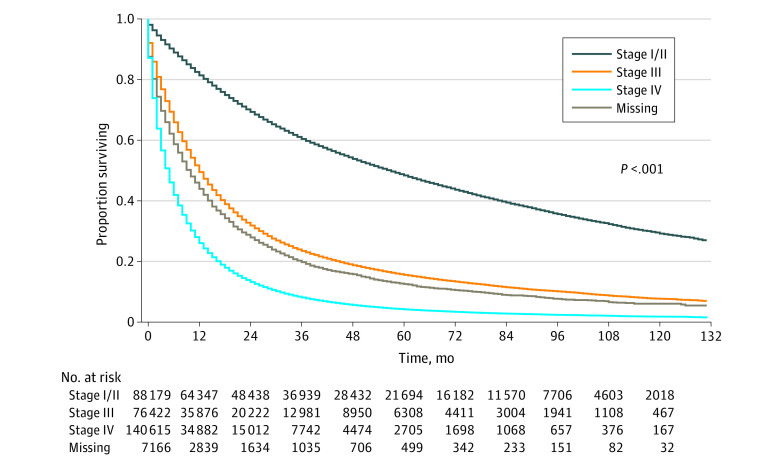
10-Year Survival According to Non–Small Cell Lung Cancer Stage at Diagnosis

Among all patients, median (IQR) follow up was 61 (21-95) months. When compared with those with known stage, those without stage information had significantly worse survival than those with stage I/II, with survival between those with stage III and stage IV (log-rank χ^2^ = 87 125.0; *P* < .001) (Figure 4). Median (IQR) survival for those with stage I/II was 57 months (18 months to not reached); for stage III it was 12 (4-34) months; for stage IV it was 5 (1-13) months; and for those missing stage it was 10 (2-28) months. Patients with stage I/II had significantly better survival compared with stage III/IV or missing stage (log-rank χ^2^ = 65 866.9; *P* < .001).

## Discussion

In this cohort study, we examined 312 382 patients in SEER diagnosed with NSCLC between 2006 and 2016 to describe trends in incidence, incidence-based mortality, stage, and histology at diagnosis. Over the last decade, lung cancer population mortality has decreased. This decline has been driven by many factors, including smoking cessation, medical therapies, CT screening, and earlier therapeutic interventions. While prior studies have explored the association of smoking cessation, earlier interventions, and targeted therapies to NSCLC mortality, the role of stage shift due to early detection has not been adequately studied.^3,4,23^ With limited precedent, this study wished to evaluate the extent of stage shift on the population level in the last decade and its association with NSCLC incidence-based mortality through a more exhaustive evaluation of stage and histology.

Consistent with prior literature, we found that the incidence-based mortality for NSCLC from 2006 to 2016 has declined at a faster rate compared with the incidence.^1,2^ To better elucidate the improvements in incidence-based mortality during the study period, we assessed the trends in stage at diagnosis. We detected a significant association between year of diagnosis and stage. Patients with stage I/II at diagnosis significantly increased (AAPC, 1.5; 95% CI, 0.5 to 2.5) from 2006 to 2016 while patients with stage III/IV at diagnosis correspondingly decreased (AAPC, −0.6; 95% CI, −1.0 to −0.2). The percentage of missing stage was relatively stable during this timeframe and did not significantly change. Moreover, we observed that of all NSCLC histologies, adenocarcinoma increased at the fastest rate from 2006 to 2016. Squamous cell carcinoma also increased during this timeframe but at a much smaller rate. Other NSCLC histologies significantly declined during this timeframe. Taken together, the increase in early stage NSCLC during the study timeframe with a corresponding increase in adenocarcinoma suggest a stage shift to earlier disease.

To corroborate these findings, we hoped to better classify patients with unknown stage to determine whether a stage shift occurred during the study timeframe or if patients with unknown stages were reclassified to more specific categories. Compared with the overall study population, those with missing stage were less likely to have adenocarcinoma and more likely to be squamous cell carcinoma and other NSCLC histologies. Furthermore, among patients without stage information, 86.7% did not undergo a surgical procedure for the primary site as part of their first course of treatment. As surgical resection is the standard of care of early stage NSCLC, the low proportion of patients with unknown stage receiving surgery seem to suggest that they might not be stage I or II at diagnosis but rather later stage.

We also examined survival rate from 2006 to 2016 to additionally define patients with unknown stage. Patients with missing stage had a median (IQR) survival of 10 (2-28) months, which was between those with stage III (12 [4-34 months]) and stage IV (5 [1-13] months). The survival rate for patients with missing stage was significantly lower compared with those with stage I/II who had a median survival of 57 months. Based on the trends in histology and survival, patients with missing stage seem to be more similar to those with stage III and IV rather than those with stage I/II. Since the proportion of patients with missing stage has remained stable over the course of 2006 to 2016, our findings support patients from later stages being shifted to earlier stages, rather than unknown stages being better classified to earlier stages.

The stage and histology shifts we describe in the SEER data are consistent on a population-level with prior studies assessing and corroborating the efficacy of low-dose CT screening for lung cancer.^24,25,26,27,28^ In particular, in the NLST, of 649 positive screening tests with low-dose CT, 70.2% were stage I and II with III and IV accounting for only 29.8%.^6^ Moreover, the majority of the early stage lung cancer tumors were indolent histologies, such as bronchioloalveolar carcinoma (BAC) and adenocarcinoma, which have an overall 5-year survival of approximately 89% and 70%, respectively, compared with 85% for stage I disease.^29,30,31,32^ In particular, 84.6% of BAC and 66% of adenocarcinoma were classified as stage I or II at the time of diagnosis.^6^ The NELSON trial further supported these results with 70.8% of participants diagnosed with stage I;^33^ 86% of adenocarcinoma and 100% of BAC were stage I or II at diagnosis with both accounting for 63.5% of early stage lung cancer with only 31.9% being late stage.^33^ Other CT-based lung cancer screening trials, including the Danish Lung Cancer Screening Trial (DLST), Italian Lung Cancer Screening Trial (ITALUNG), DANTE, Multicentric Italian Lung Detection (MILD), and German Lung cancer Screening Intervention (LUSI) have described similar stage and histology results as presented by NLST and the NELSON trial.^24,25,26,27,28^

Taken together, the increase in early-stage NSCLC with a corresponding increase in adenocarcinoma that we detected in the SEER data mirrors what prior clinical trials on CT- based lung cancer screening have described on a smaller scale. Our findings in context with these prior studies seem to suggest that awareness of CT lung cancer screening is associated with an earlier detection of NSCLC (back-alley CT screening). The greater decline in incidence-based mortality compared with the incidence of NSCLC over the past decade may be partially explained by stage and histology shifts. We realize that patient adherence to lung cancer screening with low-dose CT remains limited.^34,35,36^ According to the National Cancer Institute (NCI), uptake of CT screening has been limited and stable since 2010, with 4.5% and 5.9% of adults aged 55 to 80 years in 2010 and 2015 respectively, who met the USPSTF criteria for lung cancer screening, received a CT scan within the prior year.^19^ Thus, we cannot only attribute the trends in NSCLC incidence and incidence-based mortality over the past decade to purposeful lung cancer screening with CT.

### Limitations

The findings from this study should be interpreted within the context of its limitations. This was a retrospective study using a database that only contained preselected demographic and clinical variables. We did not have available data for smoking status, family history of lung and/or bronchial cancer, occupational exposure to carcinogens, and driver variations, such as *EGFR*, which would provide increased insight, especially when evaluating the trends in incidence-based mortality. Moreover, for the participants in the SEER database, we lack detailed information on the diagnostic method, thus limiting us from measuring the rate of CT scan uptake among the study population. Additionally, 2.3% of study population were either unstaged or their staging information was unknown. Although we performed an exhaustive analysis to better define these participants, we realize that the lack of staging for this portion of study population prevents us from recategorizing to a well-defined stage.

## Conclusions

This cohort study found that population-level mortality for NSCLC has decreased from 2006 to 2016. Although advances in treatments, particularly targeted therapeutics, have played a role in affecting mortality, our analysis suggests that decreased mortality is also associated with a diagnostic shift from later to earlier stage lung cancer and a histology shift to adenocarcinoma. Studies investigating the population impact of treatment on lung cancer mortality must take into account the confounding association of stage shift with survival and mortality outcome.
